# A Comprehensive Analysis of Mental Health Problems in India and the Role of Mental Asylums

**DOI:** 10.7759/cureus.42559

**Published:** 2023-07-27

**Authors:** Vanee R Meghrajani, Manvi Marathe, Ritika Sharma, Ashwini Potdukhe, Mayur B Wanjari, Avinash B Taksande

**Affiliations:** 1 Community Medicine, Jawaharlal Nehru Medical College, Datta Meghe Institute of Higher Education and Research, Wardha, IND; 2 Medicine, Jawaharlal Nehru Medical College, Datta Meghe Institute of Higher Education and Research, Wardha, IND; 3 Medicine and Surgery, Jawaharlal Nehru Medical College, Datta Meghe Institute of Higher Education and Research, Wardha, IND; 4 Medical Surgical Nursing, Smt. Radhikabai Meghe Memorial College of Nursing, Datta Meghe Institute of Higher Education and Research, Wardha, IND; 5 Research Scientist, Department of Research and Development, Jawaharlal Nehru Medical College, Datta Meghe Institute of Higher Education and Research, Wardha, IND; 6 Physiology, Jawaharlal Nehru Medical College, Datta Meghe Institute of Higher Education and Research, Wardha, IND

**Keywords:** community participation, policy reforms, access to care, challenges, india, mental health

## Abstract

This review article provides a comprehensive overview of the current state of mental health in India, highlighting the challenges faced, the existing initiatives, and the future directions for improving mental healthcare delivery. India is grappling with a high prevalence of mental health disorders, including depression, anxiety disorders, bipolar disorder, schizophrenia, and substance use disorders. The burden of mental health issues on individuals, families, and society is immense, leading to reduced quality of life, impaired functioning, and significant economic and social consequences. Various social and cultural factors, such as stigma, discrimination, gender inequalities, poverty, rapid urbanization, and cultural beliefs surrounding mental illness, further exacerbate the challenges of addressing mental health problems. Access to mental healthcare remains a significant concern, with considerable gaps in access to and quality of treatment and limited availability of mental health professionals, especially in rural areas. Inadequate infrastructure, a lack of awareness, and insufficient integration into primary healthcare systems hinder access to appropriate care. The historical development of mental asylums in India is examined, highlighting their establishment, purpose, and evolution over time. Critiques and challenges associated with mental asylums are discussed, including stigmatization, human rights concerns, the absence of human center approaches, quality of care, and the need for alternative approaches to mental healthcare.

## Introduction and background

With its vast population and diverse demographics, India confronts a substantial mental health burden that warrants urgent attention [1]. Mental disorders cut across various age groups, socioeconomic backgrounds, and geographical regions, impacting individuals from all walks of life [1]. The repercussions of these conditions encompass personal suffering, impaired daily functioning, and extensive societal costs [1]. The prevalence of mental health disorders in India has risen steadily in recent years, contributing to the escalating public health concern. Estimates suggest that nearly 15% of the Indian population grapples with some form of mental health issue. This figure encompasses many disorders, including anxiety disorders, depression, bipolar disorder, schizophrenia, substance use disorders, and neurodevelopmental disorders [2].

The consequences of these mental health challenges reverberate throughout society. Firstly, individuals struggling with mental health problems face immense personal anguish and distress, as these conditions often impede their ability to lead fulfilling lives. They may encounter difficulties maintaining relationships, pursuing education or employment opportunities, and participating in social activities [3]. Moreover, mental health problems substantially affect the overall functioning of communities and the nation. Decreased productivity, both in the workplace and within households, is a significant economic consequence. Mental health issues often lead to absenteeism, reduced work efficiency, and long-term disability, negatively impacting workforce productivity and economic growth [4,5].

The financial burden associated with mental health problems cannot be overlooked. Increased healthcare expenditure is incurred due to the need for mental healthcare services, including diagnosis, treatment, medication, and therapy [6]. The indirect costs, such as lost productivity and an increased burden on caregivers, further exacerbate the economic impact [6]. Beyond the economic aspect, mental health problems in India also have far-reaching social implications. Stigma and discrimination surrounding mental disorders persist in many communities, hindering individuals from seeking help and support. This leads to delays in diagnosis and treatment, perpetuating the cycle of suffering and exacerbating the long-term consequences [7].

The rising prevalence of mental health disorders in India and their multifaceted impacts necessitate a comprehensive understanding of the challenges. Addressing mental health issues becomes crucial not only for the well-being of affected individuals but also for the overall progress and development of the nation. By investigating the role of mental asylums in this context, this review article aims to shed light on potential strategies to tackle mental health problems and improve the lives of individuals grappling with these conditions in India [1,2,6]. This review article aims to comprehensively analyze mental health problems in India and explore the role of mental asylums in addressing these challenges. By examining the historical and current context, this review aims to shed light on the strengths, limitations, and potential future directions of mental asylums in the Indian mental health landscape.

## Review

Methodology

The literature search strategy involved a comprehensive approach to identifying relevant studies on mental health problems in India. Multiple databases, including PubMed, PsycINFO, and Google Scholar, were searched using a combination of keywords such as "mental health," "India," "prevalence," "burden," "access to care," and "mental health disorders." The search was conducted without any language or date restrictions to ensure the inclusion of a wide range of studies. In addition to academic literature, reports from government agencies, international organizations, and non-governmental organizations were reviewed to capture a holistic understanding of the topic. To ensure the selection of appropriate studies, specific inclusion and exclusion criteria were applied. Inclusion criteria included studies on mental health problems in India, prevalence rates, types of mental health disorders, access to mental healthcare, social and cultural factors influencing mental health, and mental health policies and initiatives in India. Both quantitative and qualitative studies were considered. Studies that provided insights into the challenges, current practices, and future directions for mental healthcare in India were prioritized. Exclusion criteria involved studies that were not specific to mental health or did not pertain to the Indian context. Studies with insufficient data, case reports, editorials, and opinion pieces were also excluded. The focus was primarily on peer-reviewed articles, systematic reviews, meta-analyses, and research reports that provided substantial evidence and analysis of mental health problems in India. The selection of studies involved a two-step process. Initially, titles and abstracts were screened to assess their relevance to the research topic. Subsequently, full-text articles were reviewed based on the inclusion and exclusion criteria. Any discrepancies or uncertainties during the study selection process were resolved through discussion and consensus among the research team members.

Mental health problems in India

Prevalence and Types of Mental Health Disorders

In India, mental health disorders have a high prevalence, impacting a considerable proportion of the population. Epidemiological studies report prevalence rates for psychiatric disorders varying from 9.5 to 370 per 1000 people in India [8]. This prevalence encompasses a broad spectrum of mental health disorders, reflecting the diverse challenges individuals face in the country [8]. The prevalence rates of mental health disorders in India highlight the need for effective interventions and support systems to address the mental well-being of the population. Conditions such as depression, anxiety disorders, bipolar disorder, schizophrenia, and substance use disorders are commonly observed mental health disorders in India [8].

Depression: Depression is a common mental health disorder characterized by persistent sadness, hopelessness, and a loss of interest or pleasure in activities. At the population level, 3.5% of deaths were attributable to anxiety or depression [9]. It can negatively impact an individual's mood, thoughts, behavior, and physical well-being. Symptoms of depression may include fatigue, changes in appetite, sleep disturbances, difficulty concentrating, and thoughts of self-harm or suicide. Depression can significantly impair a person's daily functioning, interpersonal relationships, and overall quality of life [9].

Anxiety disorders: Anxiety disorders are characterized by excessive and persistent worry, fear, or anxiety that significantly interfere with daily functioning. Generalized anxiety disorder involves chronic and excessive worry about various aspects of life. Panic disorder is characterized by recurrent panic attacks, which are intense periods of overwhelming fear and physical symptoms such as heart palpitations and shortness of breath. Phobias involve an intense fear of specific objects, situations, or activities. Obsessive-compulsive disorder (OCD) is characterized by intrusive thoughts (obsessions) and repetitive behaviors (compulsions) performed to alleviate anxiety. Anxiety disorders can cause significant distress, avoidance behaviors, and impaired functioning [10].

Bipolar disorder: Bipolar disorder is characterized by alternating periods of elevated mood (mania or hypomania) and episodes of depression. During manic episodes, individuals may experience heightened energy levels, decreased sleep, racing thoughts, inflated self-esteem, impulsive behavior, and an exaggerated sense of self-importance. Depressive episodes are marked by sadness, loss of interest, fatigue, and changes in appetite and sleep patterns. Bipolar disorder can profoundly impact an individual's emotions, behavior, relationships, and overall functioning [11].

Schizophrenia: Schizophrenia is a chronic and severe mental disorder that affects a person's perception of reality, thinking processes, emotions, and behavior. Common symptoms include hallucinations (perceiving things that are not there), delusions (false beliefs), disorganized speech and behavior, reduced emotional expression, and social withdrawal. Individuals with schizophrenia may experience difficulties in cognitive functioning, such as problems with memory, attention, and executive functioning. Schizophrenia can significantly impair an individual's ability to think, interact with others, and function in society [12].

Substance use disorders: Substance use disorders involve the excessive and compulsive use of substances, such as alcohol or drugs, despite negative consequences. These disorders can have significant impacts on mental health. Substance abuse can lead to addiction, dependence, and withdrawal symptoms when the substance is unavailable. Substance use disorders can cause various mental health issues, including mood disorders, anxiety disorders, psychosis, cognitive impairments, and social and occupational problems. The associated problems may include financial difficulties, legal issues, relationship conflicts, and physical health complications [13].

Social and cultural factors influencing mental health in India

Societal Stigma and Discrimination

Mental illness carries a significant social stigma in Indian society, leading to discrimination and social exclusion for individuals with mental health problems. The stigma surrounding mental illness often stems from misconceptions, fear, and a lack of awareness. This stigma creates barriers to seeking help and support, as individuals may fear judgment, rejection, or negative consequences. Consequently, individuals may delay or avoid seeking treatment, resulting in inadequate or delayed care and further exacerbating their condition [14].

Gender Inequalities

Gender inequalities in India have a profound impact on mental health. Women, in particular, face unique challenges and are more vulnerable to mental health problems. Factors such as domestic violence, sexual abuse, unequal power dynamics, limited access to education and employment opportunities, and societal expectations can contribute to increased stress, anxiety, and depression among women. The intersectionality of gender with other factors, such as socioeconomic status and caste, further compounds mental health disparities [15].

Poverty and Socioeconomic Factors

Poverty and socioeconomic disparities play a crucial role in developing and exacerbating mental health disorders in India. Limited resources, including access to quality healthcare, mental health services, and essential social support systems, significantly impact mental well-being. Stressful living conditions, financial instability, and a lack of opportunities for upward mobility contribute to heightened psychological distress and the risk of mental health problems [16].

Rapid Urbanization and Migration

India's rapid urbanization and migration patterns have significant implications for mental health. Urban areas often present challenges such as social dislocation, loss of social support networks, increased competition, and higher stress levels. The migration process, whether from rural to urban areas or within urban areas, can disrupt social cohesion, traditional support systems, and stability, leading to an increased risk of mental health problems [17].

Family Dynamics and Societal Pressure

Family dynamics and societal expectations pressure individuals, impacting their mental well-being. Expectations related to education, career success, marriage, and gender roles can create significant stress and anxiety. Interpersonal conflicts, strained relationships, and dysfunctional family dynamics can also contribute to developing mental health issues. In some cases, the stigma associated with mental illness within families can lead to a lack of understanding and support, further hindering the individual's ability to seek help [18].

Cultural Beliefs Surrounding Mental Illness

Cultural beliefs and traditional practices related to mental illness vary across different regions and communities in India. These beliefs can influence help-seeking behaviors, treatment approaches, and perceptions of mental health. Sometimes, cultural beliefs may stigmatize mental illness, discourage open discussions, and promote harmful practices or ineffective remedies. This can hinder access to evidence-based care and perpetuate the cycle of mental health-related challenges [19].

The Burden of Mental Health Issues on Individuals and Society

Mental health problems substantially burden individuals and society as a whole in India. Individuals with mental health disorders often experience a reduced quality of life, impaired functioning in various domains (such as work, relationships, and education), and an increased risk of suicide [20]. Societally, mental health problems lead to significant productivity losses due to absenteeism, decreased work performance, and disability. The economic impact includes increased healthcare costs and decreased productivity, impeding social and economic development [21]. Additionally, mental health problems contribute to the overall burden on the healthcare system, straining resources and diverting attention from other areas of healthcare.

Access to mental healthcare in India

Shortage of Mental Health Professionals

The availability of mental health professionals, including psychiatrists, psychologists, and psychiatric nurses, is insufficient to meet the growing demand for mental healthcare in India. The shortage is particularly prominent in rural areas, where access to mental health professionals is limited. This uneven distribution of services creates a significant barrier for individuals seeking timely and appropriate mental healthcare [22-23].

Inadequate Infrastructure and Resources

Mental healthcare facilities, especially in rural areas, often lack the necessary infrastructure, equipment, and resources to provide comprehensive care. There is a shortage of psychiatric hospitals, outpatient clinics, and community-based services. The lack of appropriate infrastructure hinders the delivery of mental healthcare services and limits the capacity to meet the diverse needs of individuals with mental health disorders [24].

Lack of Awareness and Stigma

Limited awareness and pervasive stigma surrounding mental health issues in India contribute to the underutilization of mental healthcare services. The stigma associated with mental illness leads to discrimination, social isolation, and prejudice against individuals seeking help. This stigma discourages individuals from openly discussing their mental health concerns and seeking timely treatment [25].

Insufficient Integration into Primary Healthcare

Mental health services are not adequately integrated into primary healthcare systems in India. This lack of integration results in a fragmented approach to mental healthcare, hindering early detection, timely intervention, and continuity of care for individuals with mental health problems. The separation of mental health from primary healthcare reinforces the notion that mental health is separate from physical health, perpetuating the treatment gap [26].

Historical development of mental asylums in India

Establishment and Purpose of Mental Asylums

Mental asylums were established in India during the colonial era, primarily under British rule. The first mental asylum in India, the Indian Lunatic Asylum, was established in 1745 in Calcutta (now Kolkata). These institutions were initially established to confine and segregate individuals with mental illness from the rest of society. The focus was primarily on custodial care, with little emphasis on therapeutic interventions [27].

The main objectives of mental asylums were to provide a secure and controlled environment for those deemed "insane" and to manage and control perceived threats posed by individuals with mental illness. Asylums were often located in remote areas away from urban centers and were designed to isolate individuals with mental illness from the general population [28].

Changes and Evolution of Mental Asylums Over Time

Over time, mental asylums in India have undergone significant changes and evolution. With advancements in medical understanding and changes in societal attitudes towards mental illness, the approach to care within mental asylums shifted from custodial confinement to a more humane and therapeutic approach [29].

In the mid-19th century, mental asylums began adopting moral treatment principles influenced by European reform movements. Moral treatment aims to provide a more humane and respectful environment for individuals with mental illness. It focused on promoting moral and spiritual development, engaging patients in meaningful activities, and creating a supportive therapeutic milieu [30].

In the 20th century, developing psychiatric research institutes and training centers in India further contributed to the evolution of mental healthcare practices. These institutions played a crucial role in advancing the understanding, diagnosis, and treatment of mental health disorders. They also provided opportunities to train mental health professionals and conduct research to improve care [31].

Role of Mental Asylums in Addressing Mental Health Problems

Mental asylums significantly addressed mental health problems in India, particularly when alternative options were limited. They provided a place of refuge for individuals with mental illness, offering shelter, basic care, and some level of treatment. The asylums acted as custodial institutions, ensuring the containment and management of individuals considered "insane" by societal standards [32].

Although mental asylums' quality of care and conditions varied widely, some asylums did strive to provide treatment and rehabilitation to their residents. Occupational therapy, recreation, and vocational training were introduced to promote functional improvement and reintegration into society. Some mental asylums also contributed to the understanding and treatment of mental health disorders through research and training initiatives [33].

However, it is important to acknowledge that mental asylums face significant criticism and challenges. Stigmatization, abuse, overcrowding, a lack of resources, and inadequate staff training were pervasive issues. These concerns led to an evaluation of the asylum model and the recognition of the need for broader reforms in mental healthcare delivery [34]. The role of mental asylums has evolved, and today, the focus is shifting towards community-based care, deinstitutionalization, and integrating mental health services into mainstream healthcare systems.

Critiques and challenges of mental asylums in India

Stigmatization and Social Attitudes Towards Mental Asylums

Mental asylums in India have historically faced stigmatization and negative societal attitudes. They have been associated with neglect, abuse, and human rights violations. The perception of mental asylums as places of confinement and isolation perpetuates the stigma surrounding mental health and hampers efforts to promote community-based care. This stigma often prevents individuals from seeking help and reinforces the idea that mental health conditions should be dealt with in isolation rather than as part of a broader community [35].

Human Rights Concerns and Ethical Considerations

Human rights concerns have been raised regarding mental asylums in India. Reports have documented overcrowding, a lack of privacy, and inadequate living conditions in some institutions. Patients' rights, including dignity, autonomy, and privacy, can be compromised in these settings. Additionally, the ethical considerations of involuntary admissions, the use of restraints, and the need for informed consent in psychiatric treatment are critical issues that must be addressed to protect individuals' rights and well-being [36].

Quality of Care and Treatment Modalities

The quality of care provided in mental asylums varies widely across India. While some institutions adhere to evidence-based treatments, rehabilitation programs, and a multidisciplinary approach, others struggle with resource constraints, inadequate staffing, and outdated practices. Using outdated treatments and over-reliance on medications without adequate psychosocial support services remain challenges within the mental asylum system. Improving the quality of care requires a focus on the training and capacity-building of mental health professionals, ensuring access to evidence-based treatments, and promoting holistic approaches that address the individual's social, psychological, and emotional needs [37].

Alternative Approaches to Mental Healthcare

The criticisms and challenges surrounding mental asylums have spurred the exploration of alternative approaches to mental healthcare in India. Community-based care has gained recognition as a more humane and effective approach that emphasizes the involvement of families, communities, and social support networks. Integrating mental health into primary healthcare settings allows for early detection, timely intervention, and holistic management of mental health problems. Other alternative approaches include mobile mental health units to reach underserved populations, telemedicine for remote consultations, and the implementation of psychosocial interventions that prioritize individual empowerment, resilience, and well-being. These alternative approaches promote a shift towards person-centered care and community support, reducing reliance on institutionalized care and enhancing India's overall mental health ecosystem [38].

Current mental health initiatives in India

Government Programs and Policies

The Government of India has implemented several programs and policies to address mental health issues. The National Mental Health Program (NMHP) is a flagship initiative to improve mental healthcare services. The program aims to provide accessible and affordable mental healthcare, promote community participation, train mental health professionals, and raise awareness about mental health. It also emphasizes integrating mental health into primary healthcare systems [39].

In addition, the Mental Healthcare Act of 2017 is significant legislation that prioritizes the rights and dignity of individuals with mental illness. It provides a legal framework for delivering mental healthcare, protects the rights of individuals with mental illness, decriminalizes suicide, and promotes community-based care [40].

Community-Based Mental Health Services

Community-based mental health services have gained prominence in India as a strategy to bridge the treatment gap and improve access to mental healthcare. These services adopt a decentralized approach, delivering mental healthcare at the community level through trained professionals. Community mental health programs involve outreach activities, awareness campaigns, counseling services, and support for individuals with mental health disorders and their families. The aim is to reduce stigma, enhance accessibility, and provide holistic care sensitive to communities' cultural context [38].

Integration of Mental Health into Primary Healthcare

Integrating mental health into primary healthcare is a key strategy to improve access to mental healthcare services. The District Mental Health Program (DMHP) is a notable initiative. The DMHP focuses on strengthening mental health services at the primary care level by training primary healthcare workers to identify and manage common mental health conditions. It involves capacity building, the provision of essential psychotropic medications, referral systems, and community-based rehabilitation services. This integration ensures that mental health is given equal importance to physical health, leading to early detection, timely intervention, and continuity of care [41].

Awareness Campaigns and Advocacy Efforts

Awareness campaigns and advocacy efforts are critical to promoting mental health literacy, reducing stigma, and raising public awareness about mental health issues. Non-governmental organizations (NGOs), mental health professionals, and community groups actively engage in advocacy, education, and destigmatization initiatives. These efforts aim to challenge stereotypes, provide accurate information about mental health, promote help-seeking behaviors, and create supportive environments for individuals with mental health disorders. Awareness campaigns often utilize various media platforms, community events, and workshops to reach a wide audience and promote positive attitudes toward mental health [42]. These current mental health initiatives in India demonstrate a multifaceted approach that combines government policies, community-based services, integration into primary healthcare, and awareness campaigns. Such comprehensive efforts are crucial in addressing the complex challenges of mental health and improving the overall mental well-being of individuals in the country.

Future directions for mental health in India

Increasing the Number of Mental Health Professionals

Addressing the shortage of mental health professionals requires a multi-pronged approach. One strategy is to increase the number of psychiatrists, psychologists, psychiatric nurses, and other mental health specialists. This can be achieved through expanded training programs that attract more individuals to the field and provide them with the necessary skills and knowledge to practice effectively. Scholarships and incentives can also be offered to encourage professionals to work in underserved areas where the shortage is more pronounced. By increasing the workforce in mental health, access to care can be improved [43].

Enhancing Training and Capacity-Building

To ensure the delivery of high-quality mental healthcare, it is crucial to provide comprehensive and specialized training to mental health professionals. This includes continuous professional development programs that keep professionals updated with the latest evidence-based practices. Professionals can provide more effective and targeted interventions by enhancing their knowledge and skills in diagnosing and treating mental health disorders. Training programs should focus on culturally sensitive approaches and address the specific needs of diverse populations [44].

Decentralizing Mental Health Services

To bridge the gap in mental healthcare between urban and rural areas, it is essential to strengthen mental healthcare infrastructure and services at the district and community levels. This involves establishing mental health facilities, outpatient clinics, and community-based services in rural and remote areas. By bringing mental health services closer to where people live, access to care can be improved, and individuals can receive timely interventions. This also helps reduce the burden on tertiary care centers and psychiatric hospitals [45].

Integrating Mental Health into Primary Healthcare

Recognizing the importance of early detection and intervention, integrating mental health services into primary healthcare settings is crucial. This integration involves training primary healthcare providers to identify and manage common mental health conditions. It also includes establishing referral systems between primary care and specialized mental health services. Individuals can receive timely support and treatment by integrating mental health into primary healthcare, and the stigma associated with seeking mental healthcare can be reduced [46].

Strengthening Referral Systems

To ensure seamless transitions between different levels of care, robust referral systems must be developed. Effective communication and coordination between primary healthcare providers, specialized mental health services, and other relevant sectors (such as education and employment) are essential. Referral systems should ensure that individuals with mental health problems receive continuous support and follow-up care as they move through different stages of their treatment journey. This helps maintain continuity of care and address individuals' holistic needs [47].

Public-Private Partnerships and Leveraging Technology

To improve mental healthcare delivery, collaborations between the public and private sectors can be fostered through public-private partnerships. Such partnerships can enhance resource allocation, capacity-building, and the development of innovative approaches to mental health. Private sector involvement can bring additional expertise and resources to complement public sector efforts. Furthermore, leveraging technology can significantly improve access to mental healthcare, particularly in remote and underserved areas. Telemedicine, mobile health applications, and online platforms can facilitate virtual consultations, remote monitoring, and self-help interventions, expanding the reach of mental health services [48].

By implementing these recommendations, India can make significant strides in improving mental healthcare delivery, addressing workforce shortages, enhancing training and capacity-building, decentralizing services, integrating mental health into primary care, strengthening referral systems, and harnessing the potential of public-private partnerships and technology. These strategies contribute to a more comprehensive and accessible mental health system that meets the diverse needs of individuals nationwide.

Policy reforms and resource allocation

Allocating Adequate Resources

Increasing budgetary allocations specifically for mental health is essential. Sufficient funds should be allocated to support infrastructure development, including the establishment of mental health facilities, outpatient clinics, and community-based services. Adequate resources are also necessary to recruit and train mental health professionals, implement training programs for primary healthcare providers, conduct research, and address mental health disparities across regions [49].

Prioritizing Mental Health in the Healthcare Agenda

Recognizing mental health as a priority area within the broader healthcare system is essential for effective reform. This involves integrating mental health into national health policies, strategic plans, and programs. Setting measurable targets and indicators for improving mental healthcare outcomes helps ensure that progress is monitored and interventions are evidence-based [50].

Developing a Robust Regulatory Framework

Establishing and implementing a comprehensive regulatory framework is crucial for ensuring quality standards, ethics, and guidelines for mental health services. This includes developing licensing and accreditation processes for mental healthcare providers, monitoring compliance with professional standards, and enforcing ethical guidelines. Additionally, monitoring and evaluation mechanisms should be in place to assess the quality and effectiveness of mental healthcare delivery, identify areas for improvement, and ensure accountability [51].

Ensuring Policy Implementation

Strengthening coordination and collaboration among government departments responsible for mental health, social welfare, education, and employment is necessary for effective policy implementation. Intersectoral collaboration facilitates a holistic approach to addressing mental health issues and ensures that policies and initiatives are coordinated. This coordination can include sharing resources, data, and expertise, as well as joint planning and monitoring of mental health programs [52].

Holistic and multidisciplinary approaches to mental health

Integrating Psychological, Social, and Biological Perspectives

Recognizing that mental health disorders have complex causes and manifestations, it is essential to adopt an integrated approach that addresses mental health's biological, psychological, and social determinants. This means acknowledging the interplay between genetic factors, brain chemistry, individual experiences, and social contexts in developing and managing mental health disorders [53].

Collaborative Care Models

Collaborative care models involve a coordinated and team-based approach to mental healthcare delivery. These models bring together multiple stakeholders, including mental health professionals, primary healthcare providers, social workers, and community organizations, to work collaboratively to address the needs of individuals with mental health disorders [54].

Promoting community participation and support systems

Engaging Community Leaders and Organizations

Collaborating with community leaders, religious and cultural organizations, and community-based groups is crucial for promoting mental health awareness, reducing stigma, and improving access to care. Community leaders and organizations have significant influence and reach within their communities. By partnering with them, it is possible to conduct awareness campaigns, organize educational events, and disseminate accurate information about mental health. This collaboration can help create supportive environments where individuals feel comfortable seeking help and accessing mental healthcare services. Community-based organizations can also play a role in identifying individuals in need of support and connecting them with appropriate resources [55].

Involving Individuals with Lived Experience

It is essential to involve individuals with personal experience with mental health problems in decision-making, service planning, and advocacy efforts. Their unique insights and perspectives can contribute to more person-centered and recovery-oriented mental healthcare services. These individuals can provide valuable input on the challenges they faced, the types of support that were helpful to them, and the gaps in existing services. Their involvement can help shape policies, programs, and interventions more responsive to the needs and preferences of individuals with mental health disorders. It also empowers them to become advocates for mental health and reduce stigma through sharing their stories and experiences [56].

Peer Support Networks and Community-Based Rehabilitation

Establishing peer support networks, self-help groups, and community-based rehabilitation programs is essential for fostering a sense of belonging and support among individuals with mental health disorders. Peer support networks provide a platform for individuals to connect, share experiences, and offer mutual support. These networks can help reduce feelings of isolation and provide a sense of community, which is particularly beneficial during recovery. Self-help groups allow individuals to share coping strategies, provide emotional support, and learn from each other's experiences. Community-based rehabilitation programs empower individuals with mental health disorders to develop skills, reintegrate into society, and participate in meaningful activities. These initiatives promote social inclusion, recovery, and well-being [57]. By implementing these recommendations, India can significantly improve its mental healthcare delivery, ensure better access and quality of care, reduce stigma, and promote holistic well-being for individuals with mental health disorders.

## Conclusions

Addressing mental health problems in India holds immense significance, considering the scale of human value impact involved. The country's population size gives added weight to the importance of tackling these barriers. It is crucial to recognize that mental health issues affect a significant portion of the population and can lead to severe consequences if left unaddressed. Therefore, concerted efforts are essential to combating these challenges effectively. Reducing the stigma surrounding mental illness is critical to addressing mental health problems in India. Stigma creates barriers that hinder individuals from seeking the necessary help and support they require. To overcome this, public awareness campaigns and educational initiatives are vital in combating stigma and promoting understanding and empathy toward those with mental health conditions. A comprehensive and compassionate approach is necessary to tackle India's complex mental health challenges. By reducing stigma, improving accessibility, enhancing the quality of services, shifting towards community-based care, protecting human rights, and integrating mental health into mainstream healthcare systems, India can make significant progress in addressing mental health issues. The benefits will extend beyond individuals, contributing to society's overall development and well-being.
